# Examining Short-Term Responses to a Long-Term Problem: RNA-Seq Analyses of Iron Deficiency Chlorosis Tolerant Soybean

**DOI:** 10.3390/ijms21103591

**Published:** 2020-05-19

**Authors:** Adrienne N. Moran Lauter, Lindsay Rutter, Dianne Cook, Jamie A. O’Rourke, Michelle A. Graham

**Affiliations:** 1 Corn Insects and Crop Genetics Research Unit, USDA-Agricultural Research Service, Ames, IA 50011, USA; Adrienne.MoranLauter@usda.gov (A.N.M.L.); Jamie.Orourke@usda.gov (J.A.O.); 2 Department of Statistics, Iowa State University, Ames, IA 50011, USA; lindsayannerutter@gmail.com; 3 Department of Econometrics and Business Statistics, Monash University, Clayton VIC 3800, Australia; dicook@monash.edu

**Keywords:** *Glycine max*, Iron Deficiency Chlorosis (IDC), iron stress, abiotic stress, transcriptomics

## Abstract

Iron deficiency chlorosis (IDC) is a global crop production problem, significantly impacting yield. However, most IDC studies have focused on model species, not agronomically important crops. Soybean is the second largest crop grown in the United States, yet the calcareous soils across most of the upper U.S. Midwest limit soybean growth and profitability. To understand early soybean iron stress responses, we conducted whole genome expression analyses (RNA-sequencing) of leaf and root tissue from the iron efficient soybean (*Glycine max*) cultivar Clark, at 30, 60 and 120 min after transfer to iron stress conditions. We identified over 10,000 differentially expressed genes (DEGs), with the number of DEGs increasing over time in leaves, but decreasing over time in roots. To investigate these responses, we clustered our expression data across time to identify suites of genes, their biological functions, and the transcription factors (TFs) that regulate their expression. These analyses reveal the hallmarks of the soybean iron stress response (iron uptake and homeostasis, defense, and DNA replication and methylation) can be detected within 30 min. Furthermore, they suggest root to shoot signaling initiates early iron stress responses representing a novel paradigm for crop stress adaptations.

## 1. Introduction

Iron Deficiency Chlorosis (IDC) limits growth of soybeans grown in calcareous soils across most of the upper U.S. Midwest. The primary symptom of IDC is interveinal chlorosis, caused by insufficient iron needed for chlorophyll production. Approximately 70% of cellular iron is accumulated in chloroplasts, where there is a high demand for iron for photosynthesis [1,2]. Yield losses due to IDC are estimated to be USD 120 million per year [3]. On-farm management recommendations for IDC are limited to choosing varieties resistant/tolerant to IDC or the application of expensive, and not highly effective, iron foliar sprays. However, IDC-prone fields are not uniform and IDC tolerant lines tend to have low yield on non-IDC prone soil, so farmers often choose to use higher yielding lines with higher seeding rates to overcome potential yield loss [4]. One goal for soybean breeders is to generate lines that are both IDC tolerant and high yielding. To do so will require better understanding of whole plant responses to IDC.

Work by Vert et al. [5] used split root experiments in Arabidopsis and expression levels of the iron uptake genes *FRO2* (*Ferric-chelate Reductase*) and *IRT (Iron-Regulated Transporter)* to demonstrate local low iron signaling in the root and long distance signaling from the shoot are needed to regulate root iron uptake. Enomoto and Goto [6] determined that long distance signaling from the shoot was generated by iron deficient leaves, with signaling strength dependent on the size of the plant. Giehl et al. [7] used the *irt1* mutant to demonstrate cross talk between local and long distance signaling. Recent work by Mendoza-Cozatl et al. [8] and Zhai et al. [9] suggests OPT3 in the shoot regulates iron uptake responses in the root. Recently, Hirayama et al. [10] identified *FE-UPTAKE-INDUCING PEPTIDE 1* (*FEP1*), encoding a short polypetide able to induce expression of several iron deficiency response genes independently from FIT. *FEP1* mutants exhibit normal iron accumulation in the roots, but defects in the shoot.

In dicots, which use Strategy I to take up iron from the soil, iron stress signaling results in increased expression of *Fe-deficiency Induced Transcription Factor* (*FIT*) in the root, which regulates *FRO*2 and *IRT* expression [11,12,13]. These steps convert ferric iron (Fe^+3^) to ferrous iron (Fe^+2^) and initiate iron uptake into the root. In addition, exudation of phenolic and flavin compounds help solubilize ferric iron and facilitate use of apoplastic Fe reserves [14]. Once iron has been taken into the plant, it is transported intracellularly by the natural resistance-associated macrophage proteins (NRAMP) family, which retrieve Fe from the vacuoles, and the Yellow Stripe-like (YSL) family, which binds iron chelates (such as iron bound to citrate or nicotianamine) [15,16,17]. However, specifics as to how the shoot signals to the roots in regulating these responses and how iron homeostasis is maintained have not been determined. Hormones are obvious candidates for long distance signaling and play an important role in many plant biological processes. Previous studies [18,19,20,21] have demonstrated positive roles for salicylic acid, ethylene, nitric oxide, and gibberellin in the iron uptake processes while cytokinins, jasmonic acid, and abscisic acid negatively regulate iron uptake [19]. Garcia et al. [18,19,20] demonstrated that ethylene and nitric oxide are necessary to induce expression of iron uptake genes. Wild et al. [20] found that gibberellin signaling fine-tuned the expression of the FIT-regulated genes involved in iron uptake. Shen et al. [21] found that iron deficiency increases salicylic acid levels, resulting in increased auxin and ethylene signaling and activation of iron uptake genes. In addition to hormones, iron ligands such citric acid, nicotianamine and glutathione, could play a role in iron deficiency signaling (for review see Gayomba et al. [22]). Understanding long distance IDC signaling will require examining gene expression in multiple tissues, during early IDC responses.

To understand how plants regulate responses to low iron, several transcriptomic studies have been conducted in soybeans and other plant species [23,24,25,26,27,28,29,30,31]. O’Rourke et al. [26] performed microarray analyses of soybean leaf samples and demonstrated that long term (14 days) iron stress in IDC tolerant lines led to the differential expression of genes with functions in iron uptake and homeostasis, defense and wounding, and DNA replication/methylation. While genes involved in abiotic and biotic stress responses have been identified in iron stress responses of other species, differential expression of genes involved in DNA replication and methylation is unique to soybean. Subsequent work by Atwood et al. [23] demonstrated that silencing of an iron stress responsive DNA replication gene resulted in massive transcriptional reprogramming of genes associated with defense, immunity, aging, death, protein modification, protein synthesis, photosynthesis and iron uptake and transport genes. Strikingly, Moran Lauter et al. [25] demonstrated that differential expression of these genes and pathways can be detected as early as one hour after iron stress in soybean.

Stein and Waters [29] and Waters et al. [31] measured gene expression in two Arabidopsis ecotypes 24 and 48 h after onset of iron stress. These ecotypes differed in the speed of their response to iron stress and in their patterns of differential gene expression between tissues. To compare the speed of the iron stress response between ecotypes, Stein and Waters [29] measured *FRO2* expression at twelve hour intervals for three days in both Kas-1 and Tsu-1. In Kas-1, *FRO2* expression significantly increased at 16 h, while in Tsu-1 *FRO2* expression was significant only after 48 h. In our previous study in soybean [25], we found homologs of *FIT* and *FRO2* were induced in roots both one and six hours after the onset of iron deficiency. These studies suggest that soybean, and perhaps other crops, respond to iron stress quickly and highlight the need for additional IDC studies in crop plants.

Our previous work examined transcriptional changes in leaves and roots one and six hours after iron stress [25]. We observed dynamic responses, with almost no overlap of gene expression between tissues or time points. While other studies suggest that genes differentially expressed at a single time point cannot be significant [24], our previous soybean research demonstrates that the same pathways, iron homeostasis, defense and DNA replication/methylation, are identified regardless of the duration of iron stress. Therefore, in this study we focused on iron stress responses at 30, 60 and 120 min, allowing us to capture early signaling cascades in leaves and roots. By clustering our data by expression over time, we can see how suites of genes, driven by specific sets of transcription factors (TFs), are coordinately regulated to control soybean’s iron deficiency response. In addition, comparing data between roots and leaves, suggests the presence of an early root to shoot signal in soybean. These analyses suggest important features of the crop iron stress responses have yet to be discovered.

## 2. Results

### 2.1. RNA-Seq Analyses

RNA was collected from three biological replicates of whole root and first trifoliate leaves at 30, 60, and 120 min after transfer to sufficient or insufficient conditions. Following RNA cleanup, samples were submitted for RNA sequencing. Following the quality control pipeline described in the Methods, 496.9 million reads (94.9%) successfully mapped to the Williams 82 reference genome sequence (Wm82.a2.v2 [32]). Sequencing reads generated by this study have been deposited in the National Center for Biotechnology Short Read Archive (NCBI SRA Bioproject accession PRJNA318409).

**Figure 1:**
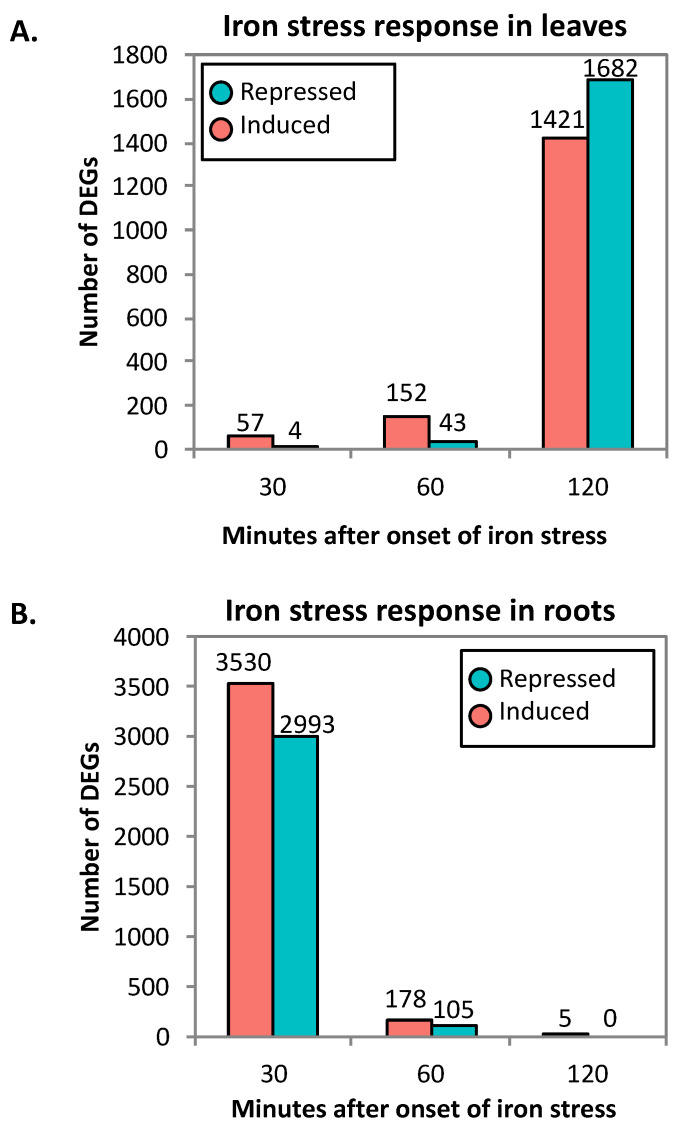
Gene expression changes dramatically in response to iron stress in leaves and roots. Significantly differentially expressed genes (DEGs) (False Discovery Rate (FDR) < 0.01) were identified by comparing gene expression in iron deficient conditions to iron sufficient conditions at each time point. (**A**) Total number of genes induced or repressed at each time point in leaves. (**B**) Total number of genes induced or repressed at each time point in roots.

To identify genes differentially expressed in response to iron stress in roots and leaves, data were normalized across time points within each tissue using TMM (trimmed mean of M-values) normalization. Statistically significant differentially expressed genes (DEGs) were identified using the edgeR statistical package [33]. While all data from a given tissue were normalized together, statistical analyses identified differentially expressed genes at each time point. Following these analyses, genes were considered significant if they had a false discovery rate (FDR) < 0.01. In the individual time points from leaves, 57 out of 61 DEGs at 30 min, 152 out of 195 DEGs at 60 min and 1421 out of 3103 DEGs at 120 min were induced by iron stress (Figure 1A, Supplemental Tables S1–S3).

This pattern of increasing number of DEGs over time in the leaves was reversed in roots. In the roots, 3530 out of 6523 DEGs at 30 min, 178 out of 283 DEGs at 60 min, and 5 of 5 DEGs at 120 min were induced by iron stress (Figure 1B, Supplemental Tables S4–S6). When comparing across time points in leaves, little overlap was observed, with 9 DEGs overlapping between 30 and 120 min and 99 DEGs overlapping between 60 and 120 min. Similarly, in roots 177 DEGs overlapped between 30 and 60 min, 1 DEG was in common between 60 and 120 min, and 2 DEGs were in common between 30 and 120 min. Between leaves and roots, there were 423 DEGs in common. Of these, only seven were significantly differentially expressed at the same time point.

### 2.2. Clustering of DEGs Based on Expression Pattern Links Gene Function, Expression and Regulation during Iron Stress

**Figure 2:**
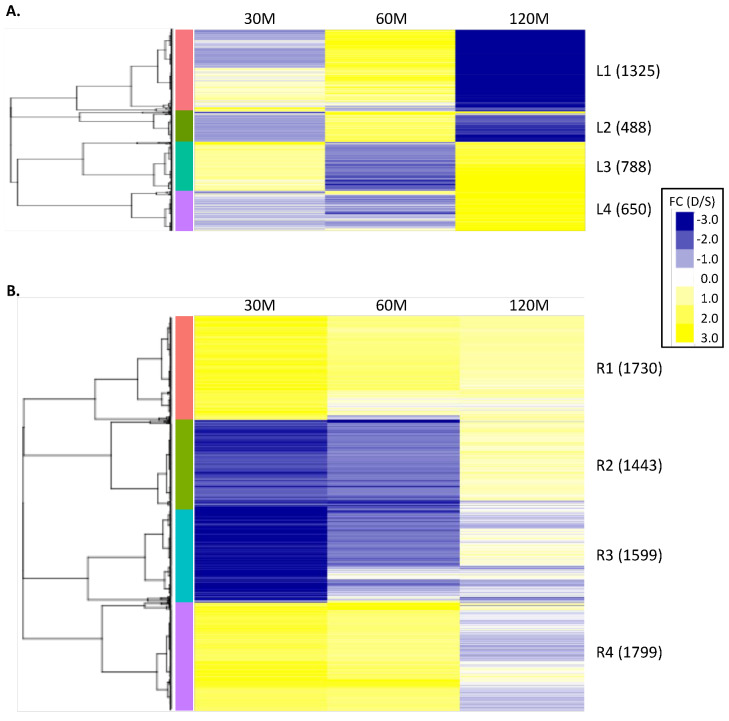
Iron stress responsive genes can be clustered by expression patterns across time. Hierarchical clustering of all fold change (FC) data was performed to characterize gene expression changes across time. Blue indicates repression and yellow indicates induction, in response to iron stress (deficient conditions/sufficient conditions) as indicated in the key. Four main clusters were indicated in leaves (**A**) and roots (**B**) across time. Total DEG number indicated in parentheses after the cluster name.

In order to understand the global transcriptional changes happening in our dataset, we performed hierarchical clustering of fold-change (FC) data on the 3251 and 6571 nonredundant iron-responsive genes from leaves and roots, respectively (Figure 2, Supplemental Tables S7 and S8). While most genes were differentially expressed at an individual time point, we included all time points to examine general expression trends and visualize the speed of gene expression changes. Within each cluster, we used a Fisher’s exact test [34] with a Bonferroni correction [35] to identify significantly overrepresented (*Probability* (*P*) < 0.05) gene ontology (GO) biological process terms [36], relative to all predicted genes in the Williams 82 reference genome. This identified biological functions associated with individual clusters. We then mined the DEG clusters for known TFs.

The 3251 non-redundant leaf DEGs grouped into four major clusters, each with a distinct expression pattern (Figure 2A, Supplemental Table S7). Cluster L1 contained 1325 DEGs whose expression was repressed or induced at 30 min (Average Fold Change, AFC30 = 0.07), induced at 60 min (AFC60 = 1.94), and then repressed again at 120 min (AFC120 = −8.25) in response to iron stress, relative to iron sufficient conditions (Figure 2). Cluster L2 (488 DEGs) had a similar expression pattern to Cluster L1, but genes were weakly repressed at 30 min and 120 min (AFC30 = −1.11, AFC60 = 5.89, AFC120 = 0.11). Clusters L3 (788 DEGs, AFC30 = 1.44, AFC60 = −2.02, AFC120 = 3.30) and L4 (650 DEGs, AFC30 = −1.02, AFC60 = −0.76, AFC120 = 6.88) also had similar expression patterns to each other, with weak expression at 30 and 60 min and strong induction in response to iron stress at 120 min.

In the roots, the 6571 non-redundant DEGs grouped into four expression clusters (Figure 2B, Supplemental Table S8). Clusters R1 (1730 DEGs, AFC30 = 2.57, AFC60 = 1.24, AFC120 = 0.94) and R4 (1799 DEGs, AFC30 = 2.23, AFC60 =1.89, AFC120 = −0.30) were strongly induced by iron stress at 30 min, weakly induced at 60 min and weakly induced or repressed by 120 min. Clusters R2 (1443 DEGs, AFC30 = −2.29, AFC60 = −1.95, AFC120 = 0.76) and R3 (1599 DEGs, AFC30 = −36.63, AFC60 = −1.26, AFC120 = −0.16) had the opposite pattern and were strongly repressed at 30 min, repressed at 60 min and either weakly induced or repressed at 120 min. In both leaves and roots, we could identify clusters that changed the direction of gene expression in as little as 30 min. In the leaves, DEGs in clusters L2 and L3 changed the direction of their expression between 30 and 60 min. Similarly, most DEGs in clusters L1 through L4 changed direction between 60 and 120 min. In the roots, DEGs in clusters R2 and R4 changed direction between 60 and 120 min. Only cluster R1 maintained the same direction of expression across time points.

**Figure 3:**
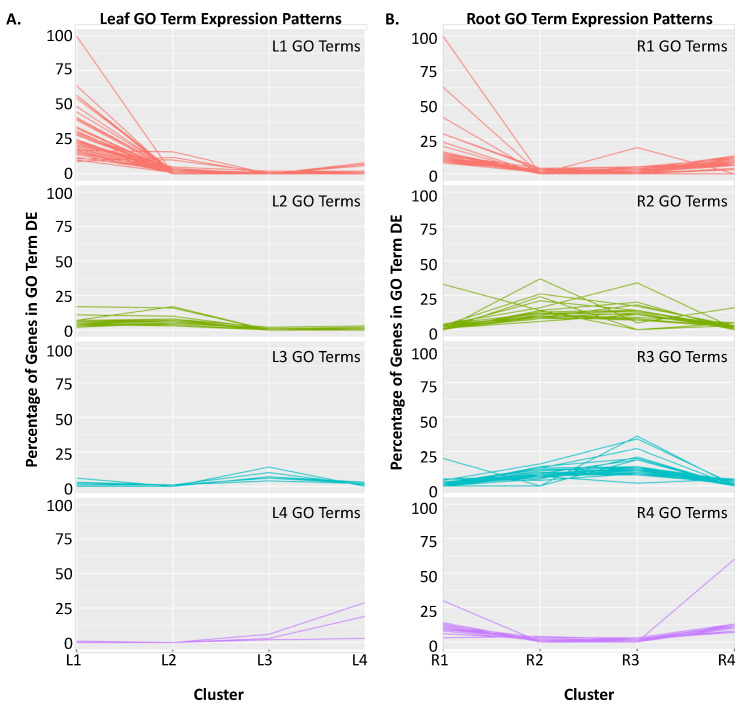
Individual gene expression clusters can be associated with specific biological functions. For each expression cluster in Figure 2 (L1–L4 and R1–R4), we identified significantly overrepresented (*p* < 0.01) GO biological process terms (Supplemental Tables S9 and S10). For each significant GO term within a cluster, we determined the number of DEGs with that GO term across all clusters. We then divided by the total number of genes in the genome with that GO term to adjust for GO terms with few genes. Note that a GO term may be significant in multiple clusters, but DEGs assigned to a GO term within a cluster are unique. (**A**) Expression patterns of leaf GO terms within leaf expression clusters. Clusters L1, L2, L3 and L4 contain 46, 16, 7 and 3 GO terms, respectively. To see the GO term descriptions for GOs assigned to each cluster, see Table 1 and Supplemental Table S9. (**B**) Expression patterns of root GO terms within root expression clusters. Clusters R1, R2, R3 and R4 contained 33, 18, 27 and 14 significant GO terms, respectively. To see the GO term descriptions for GOs assigned to each cluster, see Table 2 and Supplemental Table S10.

For each of the clusters, we then determined which GO terms were significantly (*p* < 0.05) overrepresented, relative to their representation within the soybean genome. To remove redundancy, significant GO terms within a cluster that had completely overlapping gene lists were mapped to the largest (most genes) significant GO term (Table 1 and Table 2, Supplemental Tables S9 and S10, for leaves and roots, respectively). Of the 72 GO terms identified in the leaves, 70 GO terms and the underlying DEGs were specific to an individual cluster. To demonstrate GO term specificity, we determined the number of DEGs associated with each significant GO term across all clusters. To adjust for small GO terms, we calculated the percentage of DEGs within a GO term relative to the total number of genes assigned to the GO term within the soybean genome. We then plotted this data by cluster (Figure 3A,B). This analysis also revealed that GO terms within a cluster had related biological functions (Supplemental Tables S9 and S10). In the leaves, Clusters L1-L4 contained 46, 16, 7 and 3 significantly overrepresented GO terms, respectively (Figure 3A,C, Supplemental Table S9). Cluster L1 was significantly overrepresented with multiple GO terms associated with cell cycle and gene silencing. Cluster L2 was significantly overrepresented with GO terms associated with the chloroplast and general development. Cluster L3 was significantly overrepresented with the GO terms calcium ion transport, carbohydrate metabolism, sterol biosynthesis, root hair elongation, response to gibberellin, syncytium formation and very long chain fatty acid metabolism. Cluster L4 was significantly overrepresented with the GO terms transmembrane transport, sulfate assimilation and cellular copper ion homeostasis.

Root clusters R1–R4 contained 33, 18, 27 and 14 significantly overrepresented GO terms, respectively (Figure 3B,C, Supplemental Table S10). A total of 74 unique GO terms were identified in the roots. Clusters R1 and R4, which were both induced by iron stress at 30 and 60 min, were overrepresented with GO terms related to plant defense and iron stress responses. Clusters R2 and R3 were overrepresented with multiple GO terms associated with cell wall biogenesis, development, photosynthesis and RNA methylation. Of the 74 unique GO terms identified in the roots, eight GO terms were significantly overrepresented in both R1 and R4, nine GO terms were significantly overrepresented in both R2 and R3 and one GO term was significantly overrepresented in both R1 and R3. Examining the number of DEGs within a GO term reveals the majority of GO terms were specific to an individual expression cluster.

Since hormones have proven to be important long-distant regulators of iron uptake, we looked at all statistically significant GO terms related to plant hormones identified by this study. Only four GO terms in roots (GO:0009697 (salicylic acid biosynthesis), GO:0009862 and GO:0009863 (salicylic acid mediated signaling) and GO:0009867 (jasmonic acid mediated signaling)) and one in leaves (GO:0009739, response to gibberellin stimulus) were significantly over represented in our data. Furthermore, it is unclear if the genes associated with these GO terms are associated with regulating iron uptake or defense pathways.

**Figure 4:**
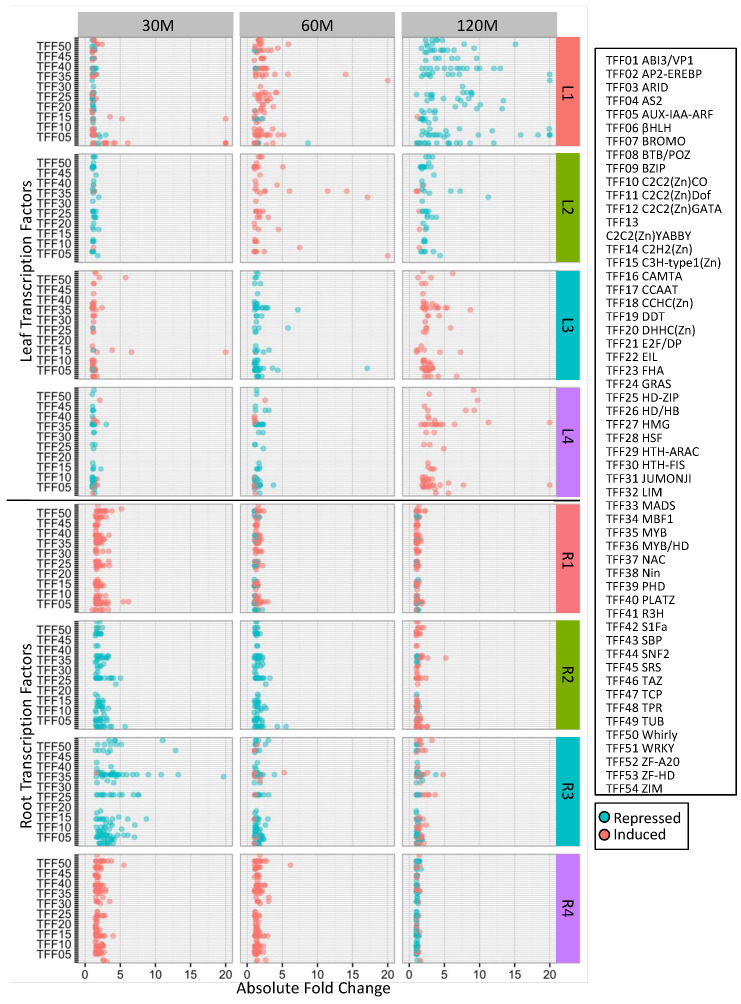
Distinct groups of transcription factors (TFs) can be associated with each iron stress responsive expression cluster. We plotted absolute fold change for each differentially expressed transcription factor by transcription factor family (TFF) within each expression cluster at each time point (Supplemental Table S11). Fold change was limited to a maximum cutoff of 20. Cluster numbers, order and color are the same as in Figure 2. Each dot represents a single transcription factor. Red dots are transcription factors induced by iron stress, while blue dots are transcription factors repressed by iron stress.

Given that DEGs clustered by their expression patterns and that clusters were associated with specific biological processes, we were interested in examining the representation and expression of TFs within each cluster (Figure 4, Supplemental Table S11). We used the SoyDB Transcription Factor Database [37] to identify all of the differentially expressed TFs within each cluster. Within clusters L1–L4, we identified 131, 56, 71 and 63 TFs, respectively. Similarly, clusters R1–R4 contained 197, 113, 137 and 181 TFs, respectively. We then examined each expression cluster to determine if particular transcription factor families (TFFs) were significantly overrepresented relative to the soybean genome. In leaves, the AUX-IAA-ARF TFF (*p* = 5.0 × 10^−3^) was overrepresented in cluster L3 (*p* = 4.0 × 10^−2^), while the ZF-HD TFF was overrepresented across all leaf DEGs (*p* < 0.01). In the roots, the WRKY (*p* = 3.5 × 10^−4^), SNF2 (*p* = 2.6 × 10^−3^) and GRAS (*p* = 5.4 × 10^−3^**)** TFFs were overrepresented in cluster R1, the HD TFF (*p* = 3.1E^−3^) was overrepresented in cluster R2, the CAMTA (*p* = 6.4 × 10^−3^) and BZIP (*p* = 1.9 × 10^−2^) TFFs were overrepresented in cluster R4, and the WRKY (*p* = 2.9 × 10^−6^), CAMTA (*p* = 2.6 × 10^−4^) and SNF2 (*p* = 3.2 × 10^−3^) TFFs were overrepresented across all root DEGs.

Of the 46 differentially expressed TFFs in leaves, only ten were observed in all four clusters (Supplemental Table S11). Of the remaining 36 families, eight were unique to cluster L1 (ABI3/VP1, C2C2(ZN)YABBY, DDT, E2F/DP, HD-ZIP, HMG, HTH-FIS and TUB), none were unique to cluster L2, two were unique to cluster L3 (HTH-ARAC and ZIM) and two were unique to cluster L4 (PLATZ and SRS). In roots, there were 49 differentially expressed TFFs, with 16 found in all four clusters. There were three TFFs unique to cluster R1 (Nin-like, TAZ and zf-A20), three unique to cluster R2 (ARID, HSF and MBF1), two unique to cluster R3 (PLATZ and ZF-HD), and three unique to cluster R4 (DDT, DHHC (Zn) and E2F/DP).

We also examined the expression patterns of the TFs themselves (Figure 4, Supplemental Table S11). As in Figure 2, while most TFs were differentially expressed at an individual time point, we included expression all time points in Figure 4 to examine general expression trends and visualize the speed of gene expression changes. Not surprisingly, the expression of the TFs within a cluster resembled the expression of all DEGs from that cluster. However, a distinct pattern could be observed when we compared TF expression in leaves and roots. In leaves, regardless of whether the TF was induced or repressed by iron stress, the magnitude of expression increased with duration of iron stress. In contrast, the magnitude of TF expression decreased with extended iron stress in the roots. Furthermore, the magnitude of gene expression was generally less than that observed in leaves.

Across time, we could observe specific TFFs differentially expressed within specific clusters. For example, in cluster L1, 12 of 16 AP2-EREBP transcription factors (TFF02, Figure 4, Supplemental Table S11) were induced by more than two-fold in response to iron stress at 30 min. By 60 min, only one family member was significantly induced more than two-fold while another was repressed. At 120 min, eight family members were significantly repressed more than two-fold. This cluster also contained 14 bHLH (TFF06), 14 PHD (TFF39) and 8 TPR (TFF48) TFs. Of the bHLH TFs, one was significantly repressed at 30 min, 7 were induced at 60 min and 13 were repressed at 120 min. For the PHD and TPR TFs, none were significantly differentially expressed at 30 min, 6 and 3 were induced, respectively, at 60 min and 13 and 8 were repressed, respectively, at 120 min. These findings suggest that for cluster L1, AP2-EREBP TFs act early at 30 min, followed by bHLH TFs at 60 min, and PHD and TPR TFs at 120 min. If we examine these same transcription factor families across all leaf clusters, we identified 0 AP2-EREBP, 5 bHLH, 2 PHD and 12 TPR TFs in cluster L2, 5 AP2-EREBP, 8 bHLH, 1 PHD and 2 TPR TFs in L3, and 2 AP2-EREBP, 10 bHLH, 1 PHD, 1 TPR TFs in L4. TFF expression differences were also evident in the root clusters.

While each of the clusters contained completely unique DEGs, when we compared clusters with similar expression patterns and biological processes, we observed differential expression of the same families of transcription factors. For example, in Clusters L3 and L4 at 60 and 120 min, we observed similar peaks for AP2-EREB (TFF02) and Myb/HD (TFF36) TFFs. Similarly, the root clusters R2 and R3 at 30 min each have peaks corresponding to the homeodomain/homeobox (TFF26) and Myb/HD (TFF36) TFFs. In contrast, cluster L1 in leaves, which was uniquely associated with the cell cycle and gene silencing, had peaks corresponding to AP2-EREBP (TFF02), bHLH (TFF06), PHD (TFF39) and TPR (TFF48) TFFs at 60 and 120 min after iron stress. This suggests that different transcription factor combinations regulate gene expression within different clusters.

## 3. Discussion

The objective of this study was to identify genes acting early in the soybean iron stress response, before a phenotype is even observed. In a previous study [25], we used RNA-seq to examine genome-wide expression changes in Clark leaves and roots in response to 1 and 6 h of iron deficiency. Given the speed and dynamic nature of the response we observed, in this study we focused on an earlier narrow window from 30 to 120 min after iron stress. In the roots, the number of iron-responsive DEGs largely diminished across time from 6523 DEGs at 30 min, to 283 DEGs at 60 min, to 5 DEGs at 120 min. In contrast, leaf DEGs increased across time from 61 DEGs at 30 min to 195 DEGs at 60 min and 3103 DEGs at 120 min.

Buckhout et al. [24] used microarray analyses to measure Arabidopsis root responses to iron stress across time (30 min, 1, 6 and 24 h). They concluded that the 90 DEGs identified at 30 min and 1 h after iron stress were not specific to the iron stress response because they were only expressed in a single time point. In our analyses we identified 3359 and 6811 iron-responsive DEGs from leaves and roots of soybean. Of these, 96.8% were specific to an individual time point. While individual DEGs were unique to specific time points, the biological processes responding to iron stress, including DNA replication, gene silencing, defense, chloroplast function and iron uptake have been reported across multiple soybean iron-response data sets [23,25,27], up to 10 days after iron stress. Furthermore, we see differential expression of homologs of canonical iron response genes such as *FER4*, *FRO6*, *NAS2*, *NAS3*, *VIT* and *YSL7* after 60 and 120 min of iron stress in the leaves (Supplemental Table S12). In the roots, homologs of *bHLH38*, *FER1*, *FER4*, *FIT*, *FRO2*, *IREG2*, *IRT3*, *NAS2*, *NAS3*, *NRAMP3*, *NRAMP6* and *YSL3* are differentially expressed after 30 min of iron stress (Supplemental Table S12). Recently, Khan et al. [38] used real-time PCR to measure gene expression of the canonical iron response genes *OPT3*, *FIT* and *IRT1* in Arabidopsis. Expression of *OPT3* was detected after 4 h of iron stress in leaves, while expression of *FIT* and *IRT1* were not detected in the roots until 8 and 12 h of iron stress, respectively. Differences observed between soybean and Arabidopsis suggest that different species have different speeds and adaptations to iron stress.

Results from other species studies support this hypothesis. Rodríguez-Celma et al. [39] used RNA-seq to compare gene expression in the root apex of Arabidopsis and *Medicago truncatula* seedlings grown in iron sufficient and deficient conditions. While Arabidopsis up-regulated the expression of genes involved in phenylpropanoid biosynthesis in response to iron stress, *M. truncatula* up-regulated the expression of genes involved in riboflavin biosynthesis. Similarly, López-Millán et al. [40] reviewed iron stress-induced metabolic adaptations made in the roots of *Beta vulgaris, Medicago truncatula*, a *Prunus dulcis* × *Prunus persica* hybrid, *Cucumis sativus* and *Solanum lycopersicum.* They found species-specific differences in the synthesis of secondary metabolites, energy-coupled transport process, and modifications to cell wall structure. Differences in iron stress responses can even be found within a species. Stein and Waters [29] and Waters et al. [31] found differences in the speed of the iron stress response and in the repertoire of differently expressed genes between two Arabidopsis ecotypes.

**Figure 5:**
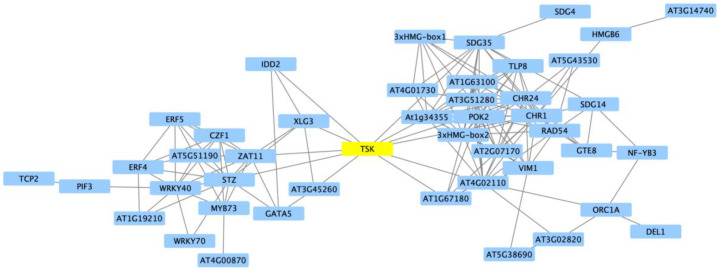
Analysis of transcription factor interactions within cluster L1 links the hallmarks of the soybean iron stress response. We used the STRING database to analyze the Arabidopsis homologs of L1 transcription factors. This analysis identified two networks of transcription factors (blue), linked by a single transcription factor, TSK (yellow). The network on the left is associated with known defense and stress TFs. The network on the right is associated with DNA replication, repair, methylation and gene silencing TFs. TSK is required for the induction of heat stress memory genes, mediates the inheritance of chromatin states across DNA replication and cell division and is associated with abiotic and biotic stress responses.

To examine the full scope of the Clark iron stress response, we combined the current data set (30, 60 and 120 min after iron stress) with previous Clark microarray and RNA-seq studies, updating all DEGs to the same version of the reference genome (Williams 82, version Wm82.a2.v2). These studies differed in expression study platform, tissues examined, age of plants and the duration of stress [25,27,41]. In total, we identified 19,870 unique genes responding to iron deficiency stress over 15 combinations of timepoints and tissues. Of these, 10,452 (52.6%) were specific to a single timepoint and tissue. Regardless of stress timing or experimental platform, the hallmarks of the Clark iron stress response have been the differential expression of genes involved in iron homeostasis, defense and DNA replication/methylation. How these pathways interconnect has remained unclear. Therefore, we leveraged the STRING database (version 10.5, [42]) to examine interactions between TFs within each cluster. Cluster L1 was significantly overrepresented with genes associated with DNA replication, chromatin modification and gene silencing (Figure 3, Supplemental Table S9). Of the 1325 DEGs in the cluster, 131 encoded predicted TFs. When we visualized the 98 unique top hit Arabidopsis homologs of the L1 TFs in STRINGDB, a striking image emerged (Figure 5, Enrichment *p*-value < 1 × 10^−16^). Two distinct networks, joined by a single TF, *TONSOKU* (*TSK,* corresponding to Glyma.05G189400 and Glyma.08G14700). Allelic mutations of TSK alter the DNA damage response, cell cycle progression, heterochromatin organization and epigenetic gene silencing [43]. Recently, Brzezinka et al. [44] demonstrated that TSK is required for the induction of heat stress memory genes and mediates the inheritance of chromatin states across DNA replication and cell division. Priming, through epigenetic modifications, has been associated with plant responses to abiotic and biotic stress (see reviews by [45,46]). Examining the top network, we see TFs involved in chromatin remodeling including CHR1/DDM1 (Glyma.01G175300 and Glyma.11G067500, [47]) and VIM1 (Glyma.02G309100, Glyma.12G001300 and Glyma.14G003700, [48]). In addition we see TFs involved in DNA replication and repair, such RAD54 (Glyma.01G244700, [49]) and POK2 (Glyma.07G209300, [50]). Examining the bottom network we see TFs involved in abiotic and biotic stress signaling including AtZAT10/STZ (Glyma.04G044900, [51]), AtZAT11 (Glyma.03G173300, [52]), AtWRKY40 (Glyma.17G222300) [53] and AtXLG3 (Glyma.09G209900, [54,55]. These findings suggest that TSK could become an important target for future crop improvement.

Given these observed differences in stress adaptations between species, and the speed and diversity of the soybean iron stress response, it makes sense to re-examine our knowledge on iron deficiency signaling. In 1996, Grusak and Pezeshgi [56], used reciprocal grafting experiments between the iron-overaccumulating pea mutant *dgl* and the parental strain to demonstrate a shoot to root signal controls root Fe(III) reductase activity in pea. Similarly, Schikora and Schmidt [57] found evidence of a shoot to root signal regulating Fe(III) reductase activity in split root experiments of wild type tomato. Vert et al. [5] used split root experiments in Arabidopsis to demonstrate that expression of *IRT1* and *FRO2* were controlled by both iron availability in the root and an unknown systemic signal from the shoot. However, it was unclear if the shoot signal was expressed in response to iron deficiency to induce root iron uptake or was expressed during iron sufficiency to repress the root iron uptake machinery. Therefore, Enomoto et al. [58] used leaf excision experiments in tobacco to demonstrate that a long distance signal regulates expression of *NtIRT1* and *NtFRO1* in the roots. Enomoto and Goto [6] found that the greater the number of excised leaves under severe iron deficient conditions, the greater the expression of *NtIRT1* and *NtFRO1,* suggesting that both plant size and iron concentration in the leaves influences iron uptake in the roots. While most of these studies have focused on dicots, recent studies have confirmed shoot to root signaling in monocot species. In wheat, application of Fe to leaves of iron-deficient plants repressed the expression of nicotianamine aminotransferase (*Ta(NAAT)*) and ferritin (*TaFER*) and prevented the release of phytosiderophores in the roots [59]. While ethylene and nitric oxide inhibitors had no effect on iron stress responses, auxin inhibitors repressed phytosiderophore release, indicating a role for auxin in controlling shoot to root signaling. Chen et al. [60] used split root experiments and shoot removal experiments to demonstrate long distance shoot to root signaling in rice.

**Figure 6:**
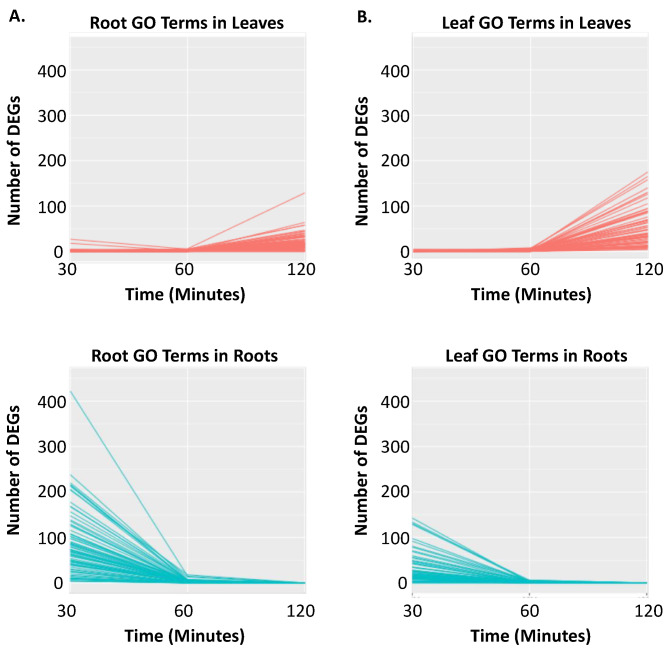
Evidence for root to shoot signaling in the soybean iron stress response. In the roots and leaves, we identified 74 and 72 unique overrepresented GO terms, respectively. To examine gene expression differences, we monitored the number of differentially expressed genes for each significant GO term across time in both roots (red) and leaves (blue). For each of the GO terms examined, differential gene expression is observed first in the roots, followed by expression later in the leaves. (**A**) Number of DEGs in significant root GO terms across tissues and time. (**B**) Number of DEGs in significant leaf GO terms across tissues and time.

However, in each of these studies, shoot to root signaling was examined several days after the onset of iron stress gene expression and much later than the iron stress response is observed in soybean. Given that so few studies have focused on time points before 24 h, we decided to survey our data for additional evidence of signaling between the distant tissues. In the roots, we identified 74 unique significantly overrepresent GO terms across the root clusters. Similarly, we identified 72 unique significantly overrepresented GO terms across the leaf clusters. Only the GO terms syncytium formation (GO:0006949, L3 and R2), rRNA processing (GO:0006364, L2 and R3) and photosystem II assembly (GO:0010207, L2 and R3) were common and significant in roots and leaves. For each significant GO term in the roots, we monitored the number of differentially expressed genes across time in the roots and the shoots. While differential gene expression associated with the significant root GO terms (Figure 6A) was shutting down in the roots after 30 min, it began to increase in the leaves between 60 and 120 min, though not enough genes were yet differentially expressed to make these GO terms significant in the leaves. Similarly, we also examined the number of DEGs in significant leaf GO terms across time and tissues (Figure 6B). Again, early expression was detected in the roots, followed by later expression in the leaves. This suggests a root to shoot signal is needed to establish iron deficiency signaling, while previous studies demonstrate a shoot to root signal is needed to maintain iron deficiency responses. Future studies are needed to determine if this response is unique to Clark, or applies to other soybean genotypes and plant species.

Multiple expression studies have demonstrated the involvement of genes associated with iron homeostasis, defense and DNA replication/methylation in the Clark iron stress response. However, mapping studies using both field conditions and hydroponics suggest a single locus controls IDC responses in Clark [61,62,63,64,65]. To study this further, we collaborated with Assefa et al. [66] to analyze a genome-wide association study for IDC tolerance in the soybean germplasm collection. The study included over 400 diverse soybean plant introduction lines from 27 countries, grown in field and hydroponic iron stress conditions. Sixty-nine genomic intervals were identified, including the major IDC tolerance QTL on soybean chromosome Gm03. By analyzing the linkage of SNPs within this QTL, the QTL could be broken into four discrete linkage blocks, each containing significant SNPs and candidate genes associated with IDC tolerance. Remarkably, eight genes differentially expressed in the present study and associated with iron homeostasis, defense and DNA replication/methylation were present within these four linkage blocks including: Glyma.03G128200 (AtTGA6), Glyma.03G128300 (AtGLU1), Glyma.03G190400 (AtBIGYIN), Glyma.03G130000 (AtRR4), Glyma.03G130400 and Glyma.03G130600 (AtBHLH38), Glyma.03G130900 (AtSDP1) and Glyma.03G131100 (AtSQD1). This suggests that genes expressed early during IDC signaling represent a molecular phenotype that can have a long-term impact on the development of a visual phenotype.

Unlike the model species Arabidopsis, soybean was domesticated more than 5000 years ago [67]. Following domestication, repeated selection by farmers and breeders likely favored changes that improved responses to a variety of stresses while selecting against changes that negatively affected yield. Furthermore, since soybean and other crop species have been adapted in multiple locations around the world, it would not be surprising if multiple stress tolerance mechanisms are present within crop germplasm collections. In this study, we focused on the soybean line Clark, an iron efficient milestone cultivar recognized by U.S. soybean breeders and producers for its contribution to yield improvements [68]. Our study highlights the speed and diversity of the soybean iron stress response and suggests new avenues for crop improvement. There is a tremendous need to study nutrient stress adaptations within agronomically important crop species. The time has come for researchers to leverage cutting edge genomics-enabled approaches and work with plant breeders to identify, characterize and utilize the genes and networks contributing to stress tolerance in crops.

## 4. Materials and Methods

### 4.1. Growth Conditions

Seeds of the soybean (*Glycine max* (L.) Merr.) line Clark (PI 548533, [69]) used in this study were originally obtained from the GRIN (Germplasm Resources Information Network) National Genetic Resources Program [70]. Seeds were increased at Bruner Farm in Ames, IA. For the current study, Clark seed was germinated on germination paper for one week in a growth chamber at Iowa State University set for 16 h light at 21 °C. Seedlings were transferred to hydroponics with iron sufficient media (100 μM Fe(NO_3_)_3_•9H_2_O) and 3% CO2 as described by Chaney et al. [71], with volumes adjusted for 10 L buckets. Chaney et al. [71] demonstrated that these nutrient solutions distinguished iron efficient and inefficient cultivars and mimicked IDC symptoms in the field. Nine days after being placed in hydroponics, the roots of all seedlings were rinsed six times in diH_2_O and transferred to either iron sufficient or deficient nutrient solutions (100 μM vs. 50 μM Fe(NO_3_)_3_•9H_2_O). Whole roots and the 1st trifoliate of plants were harvested at 30 min, 60 min and 120 min after transfer into the separate iron conditions and flash frozen in liquid nitrogen. Four biological replicates were harvested for each sample. As samples were collected before the onset of IDC, companion control plants were grown to verify expected IDC symptoms in iron-deficient conditions (data not shown).

### 4.2. RNA Isolation

Flash frozen tissue was ground in liquid nitrogen with a mortar and pestle. RNA was extracted using a Qiagen^®^ RNeasy^®^ Plant Mini Kit (Qiagen^®^, Germantown, MD, USA). The manufacturer’s protocol was followed using ~300 mg of ground tissue which was lysed using the RLT buffer and tubes were incubated at 56 °C for two min with 800 rpm shaking to aid in tissue disruption. RNA was treated with an Ambion^®^ TURBO DNA-free^TM^ kit (Ambion^®^, Austin, TX, USA) to remove all contaminating DNA. RNA quality was analyzed using an Agilent^®^ 2100 Bioanalyzer TM (Agilent^®^, Santa Clara, CA, USA). RNA was considered to be of good quality if the RNA integrity number (RIN) was greater than 7. Of the four biological replicates for each sample, the three replicates with the highest RIN values were selected for RNA-seq analyses.

### 4.3. RNA-Seq and Data Analysis

Library preparation and sequencing was performed at the Iowa State University DNA Facility using the Illumina HiSeq 2500 platform (Illumina^®^, San Diego, CA, USA). Libraries were prepared simultaneously and biological replicates were spread across lanes in the same flow cell. In total, 36 multiplex libraries were prepared from three biological replicates of the 12 samples (30, 60 and 120 min samples of roots grown in sufficient or deficient iron conditions and leaves grown in sufficient or deficient iron conditions). Library preparation and sequencing was successful for all but one library. The 100 base pair reads were trimmed prior to alignment to remove adaptor sequences (Scythe, [72]), sequencing artifacts (FASTX trimmer, [73]), and low quality bases (Sickle, [74]). Reads were then aligned to version 2 of the Williams 82 reference genome sequence (Wm82.a2.v2, [32]) using TopHat version 2.0.13 [75]. Unreliably mapped reads were removed using Samtools [76] and the resulting mapping files (bam files) were imported into the statistical program R [77] using Rsamtools [78] for statistical analysis. The Bioconductor package rtracklayer [79] was used to import the gene feature file corresponding to *G. max* version 2.0 [32] and GenomicRanges [80] was used to count reads and output a matrix containing gene counts for each sample. Only genes with log2 counts per million (cpm) > 1 in at least two replicates were used in downstream analysis.

For each tissue (leaves or roots), data were normalized using the Bioconductor package edgeR [33,81,82,83] to generate Trimmed Mean of M-values (TMM) [84]. Following normalization, the R graphics package ggplot2 [85] was used to compare sample replicates for technical reproducibility [86]. edgeR [33,81,82,87] was then used to identify DEGs at each time point. Differential expression compared iron deficient conditions to iron sufficient conditions (D/S). Following visual assessment, DE genes were considered significant with a FDR < 0.01.

### 4.4. Gene Annotation

DEGs were annotated using the SoyBase Genome Annotation Report page [88]. In brief, *G. max* primary proteins from version 2.0 were compared to all predicted proteins from the *A. thaliana* genome (The Arabidopsis Information Resource version 10) using BLASTP (E < 10^−6^, [89]). Custom Perl scripts were used to assign GO biological process terms [36] to each *G. max* protein based on the top *A. thaliana* hit. Transcription factors (TFs) were identified using the SoyDB TF database [37]. The gene identifiers of TFs present in the database were updated to reference the new genome assembly and annotation (Wm82.a2.v2, [32]).

### 4.5. Hierarchical Clustering and Heat Maps

In order to visualize responses to iron deficiency over time, we gathered the expression data for all significant DEGs across all time points within each tissue. We then performed hierarchical clustering in R [77] using hclust [90,91] within the stats package. Heat maps of clustered fold change expression data were generated using ggplot2 [85].

### 4.6. Identification of Overrepresented Gene Ontology Terms and Transcription Factor Families

Significantly overrepresented biological process GO Terms were identified using a Fisher’s exact test (1966) with a Bonferroni (1935) correction to identify significantly overrepresented GO terms within a list of DEGs relative to all genes in the soybean genome [92,93]. To remove redundancy, custom Perl scripts were used to identify any significant GO terms with completely overlapping DEGs. If identified, the larger (more DEGs) GO term is reported. A Fisher’s exact test [34] with a Bonferroni correction [35] was also used to identify overrepresented TFFs.

## Figures and Tables

**Table 1:** Top ten Gene Ontology (GO) Biological Process terms significantly overrepresented in leaf expression clusters (L1–L4).

Cluster	GO Term ID	GO Term Description	Genome Count	DEG Count	Corrected *p*-Value
L1	GO:0008283	Cell proliferation	388	161	5.64 × 10^−113^
L1	GO:0006275	Regulation of DNA replication	255	124	3.41 × 10^−97^
L1	GO:0006260	DNA replication	270	121	2.05 × 10^−89^
L1	GO:0051567	Histone H3-K9 methylation	443	148	7.86 × 10^−88^
L1	GO:0006270	DNA replication initiation	159	91	1.45 × 10^−79^
L1	GO:0000911	Cytokinesis by cell plate formation	471	139	2.16 × 10^−74^
L1	GO:0010389	Regulation of G2/M transition of mitotic cell cycle	155	85	4.88 × 10^−72^
L1	GO:0006306	DNA methylation	421	124	4.27 × 10^−66^
L1	GO:0051726	Regulation of cell cycle	345	113	1.19 × 10^−65^
L1	GO:0006334	Nucleosome assembly	128	73	2.18 × 10^−63^
L2	GO:0009658	Chloroplast organization	316	31	1.09 × 10^−13^
L2	GO:0006364	rRNA processing	532	38	1.71 × 10^−12^
L2	GO:0019288	Isopentenyl diphosphate biosynthetic process, mevalonat x 10-independent pathway	581	39	5.56 × 10^−12^
L2	GO:0045036	Protein targeting to chloroplast	114	18	6.05 × 10^−11^
L2	GO:0042793	Transcription from plastid promoter	169	21	7.32 × 10^−11^
L2	GO:0010027	Thylakoid membrane organization	469	29	9.76 × 10^−8^
L2	GO:0016226	Iron-sulfur cluster assembly	227	19	1.02 × 10^−6^
L2	GO:0045893	Positive regulation of transcription, DNA-dependent	1080	41	3.97 × 10^−5^
L2	GO:0009902	Chloroplast relocation	230	16	2.44 × 10^−4^
L2	GO:0006655	Phosphatidylglycerol biosynthetic process	176	14	2.56 × 10^−4^
L3	GO:0048767	Root hair elongation	504	31	1.30 × 10^−5^
L3	GO:0016126	Sterol biosynthetic process	363	23	4.87 × 10^−4^
L3	GO:0006949	Syncytium formation	63	9	1.92 × 10^−3^
L3	GO:0009739	Response to gibberellin stimulus	250	17	5.06 × 10^−3^
L3	GO:0006816	Calcium ion transport	324	19	1.25 × 10^−2^
L3	GO:0005975	Carbohydrate metabolic process	911	37	1.62 × 10^−2^
L3	GO:0000038	Very long-chain fatty acid metabolic process	104	10	2.00 × 10^−2^
L4	GO:0006878	Cellular copper ion homeostasis	17	5	2.19 × 10^−3^
L4	GO:0000103	Sulfate assimilation	32	6	3.91 × 10^−3^
L4	GO:0055085	Transmembrane transport	1061	36	4.39 × 10^−3^

**Table 2:** Top ten GO Biological Process terms significantly overrepresented in root expression clusters (R1–R4).

Cluster	GO Term ID	GO Term Description	Genome Count	DEG Count	Corrected *p*-Value
R1	GO:0043069	Negative regulation of programmed cell death	525	76	7.92 × 10^−14^
R1	GO:0002679	Respiratory burst involved in defense response	420	61	6.99 × 10^−11^
R1	GO:0031348	Negative regulation of defense response	766	89	1.52 × 10^−10^
R1	GO:0009863	Salicylic acid mediated signaling pathway	458	62	1.04 × 10^−9^
R1	GO:0050832	Defense response to fungus	941	100	1.40 × 10^−9^
R1	GO:0000165	MAPK cascade	575	70	6.40 × 10^−9^
R1	GO:0010363	Regulation of plant-type hypersensitive response	1019	103	1.23 × 10^−8^
R1	GO:0009862	Systemic acquired resistance, salicylic acid mediated	688	77	4.19 × 10^−8^
R1	GO:0006612	Protein targeting to membrane	1020	101	6.11 × 10^−8^
R1	GO:0009697	Salicylic acid biosynthetic process	653	73	1.50 × 10^−7^
R2	GO:0006412	Translation	763	100	6.94 × 10^−29^
R2	GO:0042254	Ribosome biogenesis	264	37	9.55 × 10^−11^
R2	GO:0001510	RNA methylation	418	46	8.74 x 10^−10^
R2	GO:0009664	Plant-type cell wall organization	337	39	9.48 × 10^−9^
R2	GO:0009834	Secondary cell wall biogenesis	135	21	2.61 × 10^−6^
R2	GO:0044036	Cell wall macromolecule metabolic process	222	27	4.35 × 10^−6^
R2	GO:0015979	Photosynthesis	452	40	1.44 × 10^−5^
R2	GO:0010089	Xylem development	225	26	2.39 × 10^−5^
R2	GO:0010014	Meristem initiation	342	33	3.20 × 10^−5^
R2	GO:0006949	Syncytium formation	63	13	8.33 × 10^−5^
R3	GO:0006412	Translation	763	110	2.38 × 10^−36^
R3	GO:0009834	Secondary cell wall biogenesis	135	46	2.17 × 10^−31^
R3	GO:0042254	Ribosome biogenesis	264	53	9.57 × 10^−24^
R3	GO:0001510	RNA methylation	418	58	1.03 × 10^−17^
R3	GO:0015979	Photosynthesis	452	54	1.66 × 10^−13^
R3	GO:0045492	Xylan biosynthetic process	497	56	6.24 × 10^−13^
R3	GO:0010207	Photosystem II assembly	372	46	7.35 × 10^−12^
R3	GO:0006364	RNA processing	532	56	1.16 × 10^−11^
R3	GO:0019684	Photosynthesis, light reaction	320	42	1.33 × 10^−11^
R3	GO:0009773	Photosynthetic electron transport, photosystem I	121	25	8.97 × 10^−11^
R4	GO:0006487	Protein N-linked glycosylation	298	40	2.65 × 10^−6^
R4	GO:0015706	Nitrate transport	486	54	7.70 × 10^−6^
R4	GO:0006468	Protein phosphorylation	2386	171	5.38 × 10^−5^
R4	GO:0009863	Salicylic acid mediated signaling pathway	458	49	9.58 × 10^−5^
R4	GO:0045087	Innate immune response	274	34	2.69 × 10^−4^
R4	GO:0006952	Defense response	1116	91	3.78 × 10^−4^
R4	GO:0010106	Cellular response to iron ion starvation	237	30	8.67 × 10^−4^
R4	GO:0071366	Cellular response to indolebutyric acid stimulus	10	6	1.98 × 10^−3^
R4	GO:0030968	Endoplasmic reticulum unfolded protein response	501	49	2.04 × 10^−3^
R4	GO:0000041	Transition metal ion transport	201	25	9.36 × 10^−3^

## Supplementary Materials

Supplementary materials can be found at https://www.mdpi.com/1422-0067/21/10/3591/s1; Supplemental l file 1: Table titles; Table S1. Genes significantly differentially expressed in leaves 30 min post iron stress; Table S2. Genes significantly differentially expressed in leaves 60 min post iron stress; Table S3. Genes significantly differentially expressed in leaves 120 min post iron stress; Table S4. Genes significantly differentially expressed in roots 30 min post iron stress; Table S5. Genes significantly differentially expressed in roots 60 min post iron stress; Table S6. Genes significantly differentially expressed in roots 120 min post iron stress; Table S7. Annotation and expression information for the leaf expression clusters L1-L4.; Table S8. Annotation and expression information for the root expression clusters R1-R4.; Table S9. Gene ontology (GO) biological process terms overrepresented in leaf DEG expression clusters (L1-L4); Table S10. Gene ontology (GO) biological process terms overrepresented in root DEG expression clusters (R1-R4); Table S11. Transcription factors differentially expressed in response to iron stress at 30, 60 and/or 120 min.; Table S12. Soybean homologs of canonical iron genes differentially expressed in response to iron stress at 30, 60 and/or 120 min in the leaves and roots.

## Abbreviations

AFC	Average Fold Change
DEGs	Differentially expressed genes
FC	Fold Change
FDR	False discovery rate
GO	Gene ontology
IDC	Iron deficiency chlorosis
P	Probability
TF	Transcription factor
TFFs	Transcription factor families
TMM	Trimmed mean of M-values
